# Teprotumumab N01 for thyroid eye disease in China: clinical outcomes and quantitative MRI correlations

**DOI:** 10.3389/fmed.2026.1805546

**Published:** 2026-03-20

**Authors:** Qiuyu Wang, Junning Cheng, Chunhui Yang, Jian Song, Yuqing Chen, Ya Shen, Lefeng Qu, Ruili Wei

**Affiliations:** 1 Department of Ophthalmology, The Second Affiliated Hospital of Naval Medical University, Shanghai, China; 2 Department of Vascular and Endovascular Surgery, The Second Affiliated Hospital of Naval Medical University, Shanghai, China; 3 Department of Ophthalmology, No. 906 Hospital of People’s Liberation Army, Ningbo, China

**Keywords:** thyroid eye disease, teprotumumab, real-world study, magnetic resonance imaging, inferior rectus

## Abstract

**Background:**

Teprotumumab is an IGF-1R–targeted therapy for thyroid eye disease (TED). In routine practice, response assessment remains challenging and standardized imaging biomarkers are lacking.

**Objective:**

To evaluate the real-world effectiveness of domestically produced teprotumumab N01 injection (SYCUME®) in Chinese patients with TED and to identify inferior rectus (IR)–focused quantitative MRI measures associated with changes in proptosis and disease activity.

**Methods:**

We conducted a retrospective real-world study of patients with active moderate-to-severe TED who completed a full course of teprotumumab N01 between March and December 2025. Orbital MRI was acquired before and after treatment. IR, lacrimal gland (LG), and orbital fat (OF) were obtained using ROI-based analysis on MAGiC (T1, T2, proton density) and IDEAL-IQ (fat fraction). MRI proptosis and coronal morphologic measures were quantified in 3D Slicer. Eye-level changes and associations between clinical outcomes and MRI parameter changes (*Δ*, post minus pre) were analyzed using linear mixed-effects models with a subject-specific random intercept, with Benjamini–Hochberg adjustment for MRI parameters. Laboratory indices and GO-QOL were analyzed at the patient level.

**Results:**

Twenty-two patients (44 orbits) were analyzed. Hertel proptosis and CAS decreased after treatment (both *p* < 0.001). MRI proptosis decreased (*p* < 0.001) with reductions in IR maximum coronal length and width (both *p* < 0.001). IR ADC decreased (*p* = 0.010). Mapping metrics showed decreases in IR T1 and proton density (both *p* < 0.001), while IR T2 did not reach statistical significance. Fat fraction increased in IR and OF. In *Δ* analyses, ΔMRI proptosis was associated with ΔIR maximum coronal length and width, and ΔCAS was associated with ΔIR morphologic changes and ΔIR ADC, with additional associations involving selected LG and OF quantitative parameters after multiple-comparison adjustment. Thyroid hormone levels remained stable overall. Selected antibodies and IGF-1 changed significantly. GO-QOL improved (all *p* < 0.001). At the patient level, only *Δ* TRAb and ΔIGF-1 were associated with ΔCAS, and no laboratory change was associated with ΔGO-QOL.

**Conclusion:**

In this real-world Chinese cohort, teprotumumab N01 was associated with concordant clinical improvement and quantitative MRI changes. IR-focused morphologic measures and diffusion-derived ADC showed the most consistent associations with changes in proptosis and disease activity, supporting their further evaluation for treatment monitoring in TED.

## 1 Introduction

Thyroid eye disease (TED), also known as thyroid-associated ophthalmopathy or Graves’ ophthalmopathy, is an autoimmune-mediated inflammatory disorder characterized by the enlargement of extraocular muscles (EOM) and orbital fat (OF). This leads to clinical manifestations such as proptosis, diplopia, motility restriction, and, in severe cases, vision impairment (1–4). The inferior rectus (IR) is most commonly affected, followed by the medial rectus (MR), superior rectus (SR), and lateral rectus (LR) muscles (3). The pathophysiology involves the activation of orbital fibroblasts through thyroid-stimulating hormone (TSH) and insulin-like growth factor-1 receptor (IGF-1R) signaling, triggering inflammation, tissue swelling, and fibrosis (2, 5).

Teprotumumab, a monoclonal antibody targeting the IGF-1R, is the first IGF-1R inhibitor approved for TED treatment. Recently, teprotumumab N01 injection (SYCUME®; “信必敏”) was approved in China as the first domestically produced IGF-1R–targeted therapy for TED. Clinical studies have demonstrated its efficacy in moderate-to-severe active TED, particularly in reducing proptosis and improving clinical activity (6). However, objective imaging-based metrics and biomarkers for monitoring treatment response remain insufficiently defined, highlighting the need for quantitative approaches that better reflect tissue-level change (7).

Magnetic resonance imaging (MRI) provides high soft-tissue contrast and multi-parametric characterization of orbital structures, and it is widely used for diagnosis, activity assessment, and follow-up in TED. Typical MRI features include EOM involvement, proptosis, OF expansion, lacrimal gland (LG) enlargement, and orbital apex crowding, which is relevant for dysthyroid optic neuropathy (7, 8). Beyond qualitative assessment, MRI can generate quantitative readouts, such as morphologic measurements, diffusion-weighted imaging (DWI)–derived metrics, and relaxation or composition-related parameters, which may capture treatment-related tissue alterations (9).

While teprotumumab has demonstrated promising clinical efficacy, assessing treatment response remains challenging. Standard clinical measures, including clinical activity score (CAS) and external proptosis measurements, are clinically useful but may not fully reflect underlying orbital tissue remodeling (10). Quantitative MRI may therefore serve as an adjunct by providing objective measures that are closer to structural and compositional changes in the orbit. At present, there is no consensus regarding which MRI-derived parameters are the most reliable for treatment monitoring, and evidence in real-world Chinese cohorts remains limited (7, 11–13).

In this retrospective real-world study, we evaluated the effectiveness of domestically produced teprotumumab (SYCUME®) in Chinese patients with TED and examined quantitative MRI measures as imaging correlates of clinical response. Given that IR involvement is common and clinically consequential in TED, we prioritized IR-focused morphologic and quantitative parameters, and assessed their associations with key outcomes including proptosis and CAS. This work aims to inform practical imaging-based monitoring strategies and to support future standardization of quantitative MRI biomarkers in TED.

## 2 Materials and methods

### 2.1 Subjects

**Figure 1:**
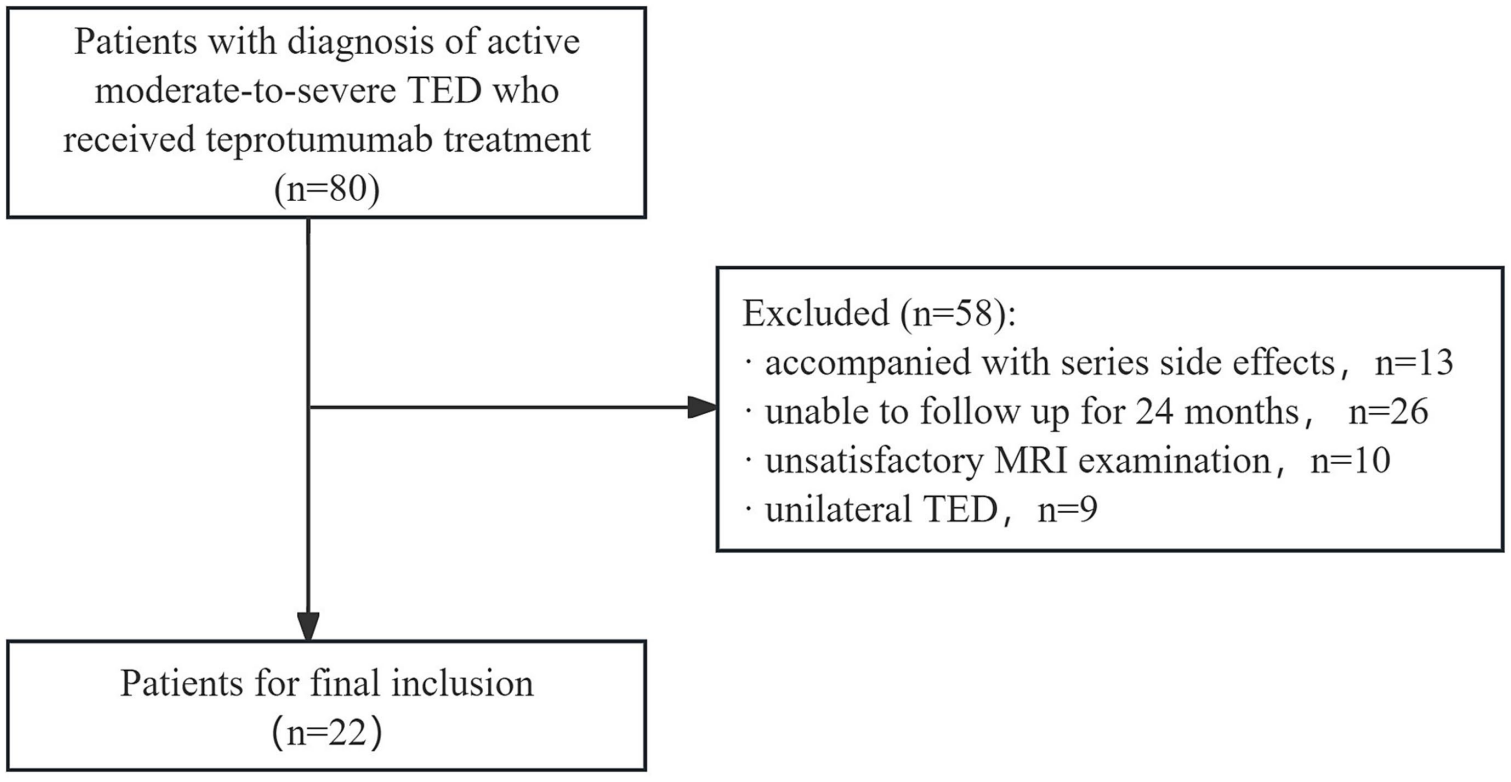
The flowchart of enrolled patients.

This retrospective study enrolled patients with TED who received a full course of teprotumumab between March and December 2025. The diagnosis and evaluation of TED was following the consensus statement of the European Group on Graves’ Orbitopathy (EUGOGO) criteria (14). Eligible patients were aged 45–80 years and weighed 45–100 kg, with active moderate-to-severe TED [Clinical Activity Score on the 7-point scale (CAS-7) ≥ 3]. Patients with prior orbital radiotherapy, prior immunomodulatory agents, or prior orbital surgery (including decompression), as well as those with concurrent orbital diseases, were excluded. A total of 80 patients were initially screened, of which 26 were excluded due to irregular follow-up, 10 had unsatisfactory MRI results, 13 had severe side effects or contraindications, and 9 had unilateral TED. Ultimately, 22 patients (44 orbits) were included in the final analysis. The flowchart of participant screening and enrollment is shown in Figure 1.

### 2.2 Ethics approval

Ethical approval for this retrospective study was obtained from the Ethics Committee of the Changzheng Hospital Biomedical Research Ethics Committee (Identifier: 2021SL044). This research adhered to the principles outlined in the Declaration of Helsinki. Due to the utilization of de-identified images and personal information, the requirement for informed consent was waived.

### 2.3 MRI imaging

Orbital MRI was performed before and after teprotumumab treatment to evaluate changes in the IR, LG, OF, and other relevant anatomical structures. The following MRI sequences were utilized: T1-weighted imaging (T1WI): Used to evaluate the morphology of the OF and IR. T2-weighted short tau inversion recovery (T2 STIR): Used to evaluate edema and inflammatory-related increases in tissue water content. DWI: Applied to assess microstructural alterations in the IR. Magnetic resonance image compilation (MAGiC): Used to assess T1 mapping (T1), T2 mapping (T2), and proton density (PD) in the IR, LG, and OF. Iterative decomposition of water and fat with echo asymmetry and least-squares estimation (IDEAL-IQ): for quantifying fat fraction in the IR, LG, and OF.

Orbital MRI was performed on a 3.0-T scanner (Achieva 3.0 T TX, Philips Healthcare, Best, Netherlands) using a 32-channel head coil. Patients lay supine and were instructed to keep their gaze steady and minimize eye movements to reduce motion-related artifacts. Imaging parameters were as follows. T1WI was acquired with repetition time (TR) 470 ms and echo time (TE) 12 ms, a slice thickness of 3 mm, an inter-slice gap of 0.3 mm, and a field of view (FOV) of 160 × 180 mm. T2WI was acquired with TR 3000 ms and TE 80 ms using the same slice thickness, inter-slice gap, and FOV. DWI was acquired with b values of 0 and 800 s/mm^2^. Quantitative sequences were acquired with similar geometric coverage, with an FOV of 160 × 180 mm for MAGiC and 180 × 180 mm for IDEAL-IQ.

**Figure 2:**
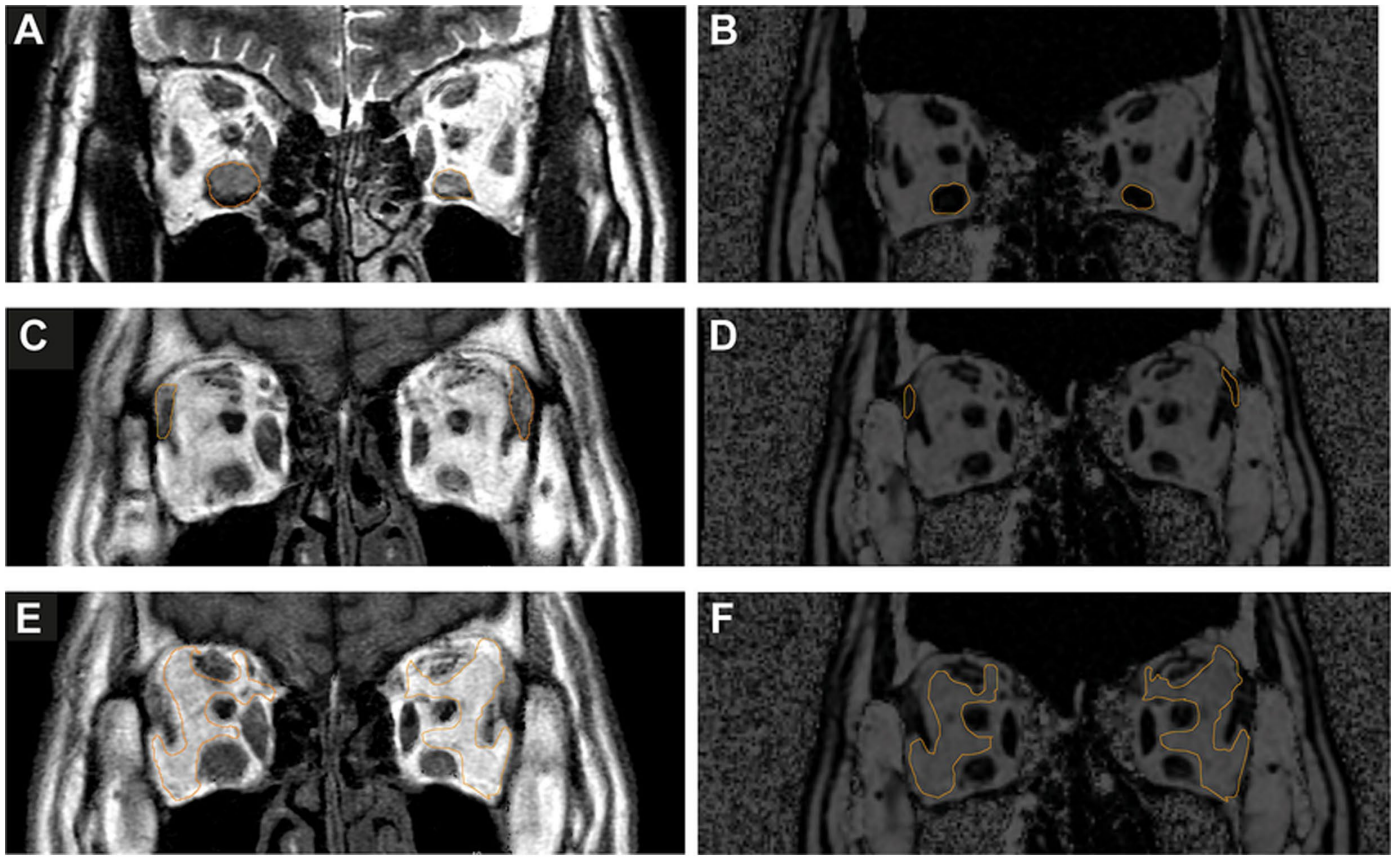
Region-of-interest (ROI) definition for quantitative MRI analysis. The ROI was placed along the perimeter of the greatest enlargement of IR, LG, and OF in a coronal section. **(A)** IR ROI on MAGiC. **(B)** IR ROI on IDEAL-IQ. **(C)** LG ROI on MAGiC. **(D)** LG ROI on IDEAL-IQ. **(E)** OF ROI on MAGiC. **(F)** OF ROI on IDEAL-IQ.

For each structure, the region of interest (ROI) was drawn along the visible outer margin to capture the largest cross-sectional area, avoiding adjacent structures and obvious vessels when present. ROIs were placed on the MAGiC images for quantitative T1, T2, and PD and on the corresponding IDEAL-IQ images for fat fraction (FF) quantification. The ROIs were places on the MAGiC images for quantitative T1, T2, and PD (Figures 2A, C, E), IDEAL-IQ images for FF quantification (Figures 2B, D, F), and apparent diffusion coefficient (ADC) maps for corresponding values. Imaging was analyzed by two independent radiologists, with disagreements resolved by consensus. The scanner software release was R5.6.1, and quantitative maps were generated using the vendor’s built-in reconstruction workstation, conflicts were resolved by discussion.

### 2.4 Imaging processing

**Figure 3:**
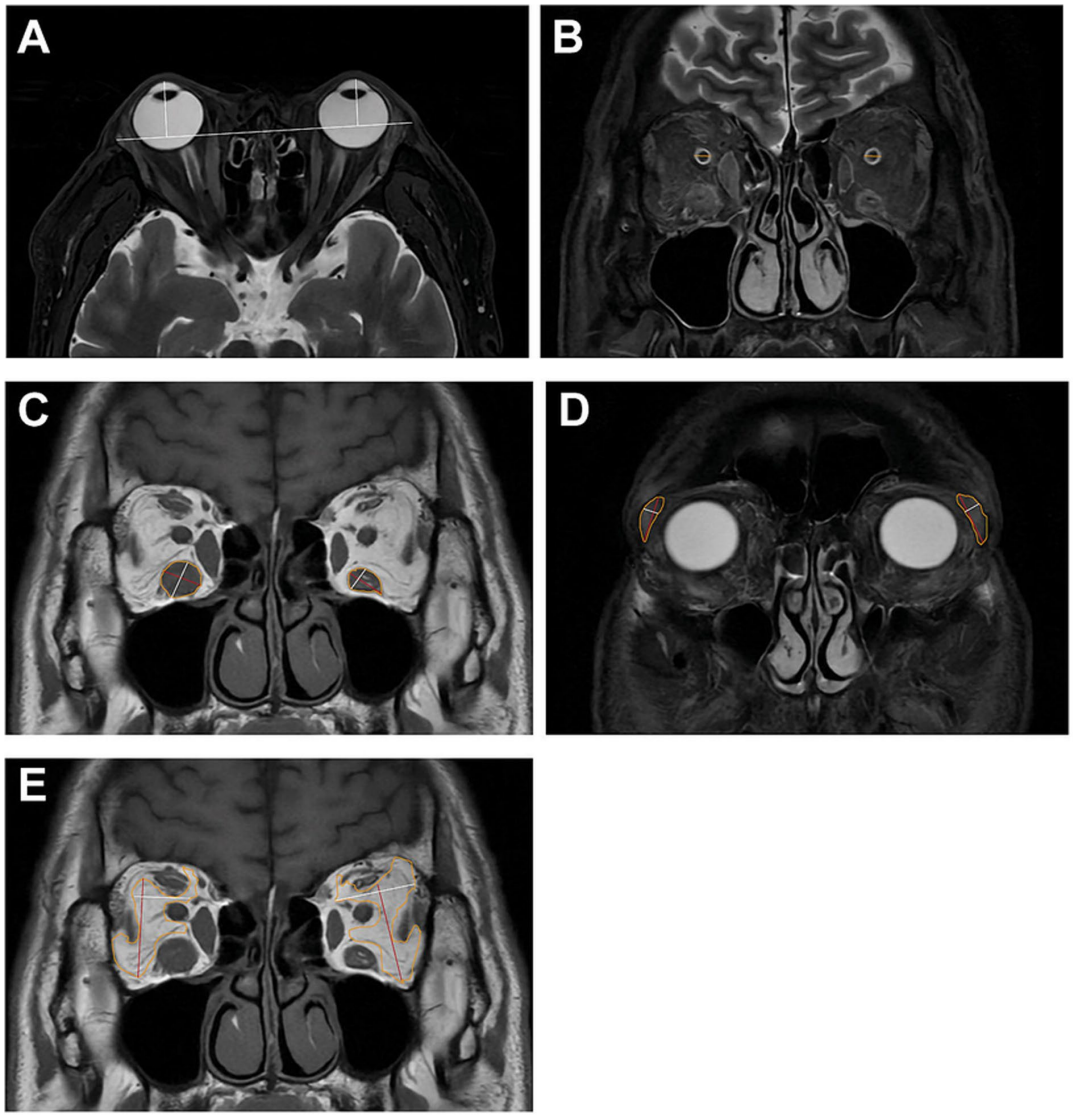
Measurement of MRI proptosis, optic nerve sheath diameter, maximum coronal length (MCL), and width (MCW) of the IR, LG, and OF. **(A)** MRI proptosis was defined as the perpendicular distance from the corneal eminence to a reference line connecting the most anterior points of the bilateral lateral orbital rims, measured on an axial T2 STIR image. **(B)** ONSD on a coronal T2 STIR image. **(C–E)** MCL (red line) and MCW (white line) of the inferior rectus (IR), lacrimal gland (LG), and orbital fat (OF).

In addition to MRI sequences, measurements including MRI proptosis, optic nerve sheath diameter (ONSD), maximum coronal length and width of the IR, LG, and OF were measured using 3D Slicer (version 5.10.1; https://www.slicer.org/) (Figure 3). Two independent specialists (QW and JC), uninformed of any information about the study population in the process of quantitative evaluation, independently performed these measurements using 3D Slicer software. If a disparity of over 10% was detected between two specialists, consensus was reached through open adjudication. Agreement between paired measurements was evaluated using Bland–Altman analysis, which revealed good agreement, with 95% of paired measures falling within the limits of agreement (LOAs); no systematic errors were observed. Then the results of observer 1 were used for further statistical analysis.

### 2.5 Clinical and laboratory biomarkers data

Extensive demographic information involving age, gender, disease course, smoking history, treatment experiences were retrieved from medical records. Additionally, Snellen best-corrected visual acuity (BCVA), intraocular pressure (IOP, Topcon, Japan), Hertel proptosis, CAS (CAS-7), quality of life (QOL), and experimental tests including total triiodothyronine (T3), total thyroxine (T4), free triiodothyronine (FT3), free thyroxine (FT4), thyroid-stimulating hormone (TSH), thyroglobulin (TG), thyroglobulin antibody (TG Ab), thyroid peroxidase antibody (TPO Ab), thyrotropin receptor antibody (TRAb), natural killer cells (NK), percentages and absolute counts of CD3 + T cells, CD4 + T helper cells, CD8 + cytotoxic T cells, and CD19 + B cells, parathyroid hormone (PTH), insulin-like growth factor 1 (IGF-1) and Graves’ orbitopathy-specific quality of life questionnaire (GO-QOL) were retrieved from medical records.

### 2.6 Statistical analysis

Data analyses were performed using R (version 4.3.3). Continuous eye-level variables are presented as mean (95% confidence interval). Pre- and post-treatment differences in eye examinations and MRI parameters were evaluated using linear mixed-effects models, with a subject-specific random intercept to account for inter-eye dependence and repeated measurements. Associations between changes (*Δ* values) and between MRI parameters were assessed with the fixed-effect t statistic, and the effect size was calculated using the r-equivalent. MRI parameter *p*-values were adjusted using the Benjamini–Hochberg false discovery rate procedure. Categorical variables are presented as counts and percentages. For continuous variables, data are presented as mean ± SD for normally distributed variables and median (Q1, Q3) for non-normally distributed variables. Paired *t*-tests were used for normally distributed variables, and Wilcoxon signed-rank tests were used for non-normally distributed variables. Spearman correlation coefficients were applied to examine the relationship between changes in laboratory measures and changes in CAS and GO-QOL. *p* values < 0.05 were considered statistically significant.

## 3 Results

A total of 22 patients (44 orbits) were included. Fifteen patients (68.2%) were female and seven (31.8%) were male. The mean age was 45.82 ± 11.00 years. The disease duration prior to teprotumumab initiation was 10.0 months (IQR, 6.25–30.75; range, 3–360). Twenty-one patients (95.5%) were never smokers and one (4.5%) was a current smoker. Nineteen patients (86.4%) had received prior thyroid treatment. None of the patients had received radiotherapy or other immunomodulatory agents. Four patients (18.2%) had received systemic glucocorticoids before teprotumumab. All baseline imaging was obtained after discontinuation of glucocorticoids. Sixteen of 22 patients (72.7%) were classified as EOM-predominant and 6 (27.3%) as proptosis-predominant. Among EOM-predominant cases, 4 of 16 (25.0%) had single-muscle involvement and 12 (75.0%) had multi-muscle involvement. In the multi-muscle subgroup (*n* = 12), the most frequently involved muscles were the IR (10/12, 83.3%), SR (7/12, 58.3%), MR (6/12, 50.0%), and LR (2/12, 16.7%). Detailed results are presented in Table 1.

**Table 1:** Demographical characteristics of patients (*n* = 22).

Group	Number (percentage)
Gender	
Male	7 (31.8%)
Female	15 (68.2%)
Age (years)	
21–30	3 (13.6%)
31–40	4 (18.2%)
41–50	5 (22.7%)
51–60	8 (36.4%)
61–70	2 (9.1%)
Disease course	
< 6 months	6 (27.3%)
Half-1 year	9 (40.9%)
1–3 years	5 (22.7%)
> 3 years	2 (9.1%)
Smoking history	
Never smoker	21 (95.5%)
Current smoker	1 (4.5%)
Treatment experiences^a^	
Treatment on thyroid	19 (86.4%)
Glucocorticoid	4 (18.2%)
Orbital radiotherapy or other immunotherapy	0 (0%)
Predominant phenotype^b^	
EOM-predominant	16 (72.7%)
Proptosis-predominant	6 (27.3%)
EOM involvement pattern in EOM-predominant patients (*n* = 16)	
Single-muscle	4 (25%)
Multi-muscle	12 (75%)
Muscles involved in multi-muscle EOM-predominant patients (*n* = 12)	
SR	7 (58.3%)
IR	10 (83.3%)
MR	6 (50.0%)
LR	2 (16.7%)

^a^Patients with TED could have received more than one prior treatment. Therefore, totals may exceed 22 and percentages may sum to >100%.

^b^EOM-predominant indicates predominant EOM enlargement, whereas proptosis-predominant indicates predominant proptosis on Hertel and MRI with no prominent EOM enlargement. Within EOM-predominant cases, muscle involvement was summarized as single- or multi-muscle, and involved muscles were recorded on baseline MRI.

### 3.1 Clinical and MRI changes after teprotumumab treatment

Pre-treatment and post-treatment findings are summarized in Table 2. Comparisons were performed using linear mixed-effects models accounting for inter-eye correlation, and *p* values for MRI parameters were adjusted using the Benjamini–Hochberg procedure.

**Table 2:** Comparison of eye examinations and MRI parameters before and after teprotumumab treatment (mean and 95% CI).

Parameters	Pre-treatment (*n* = 44 orbits)	Post-treatment (*n* = 44 orbits)	*p*-value
Eye examinations			
Hertel proptosis (mm)	18.78 (17.61, 19.95)	16.85 (15.68, 18.02)	*p* < 0.001^***^
CAS	2.80 (2.33, 3.26)	1.41 (0.94, 1.88)	*p* < 0.001^***^
BCVA (logMAR)	0.02 (0.00, 0.04)	0.00 (−0.02, 0.02)	*p* = 0.055
IOP (mmHg)	13.75 (12.73, 14.78)	13.87 (12.85, 14.89)	*p* = 0.789
MRI parameters			
MRI proptosis (mm)	21.69 (20.51, 22.88)	18.88 (17.69, 20.06)	*p* < 0.001^***^
Optic nerve sheath width (mm)	3.39 (3.08, 3.69)	3.82 (3.51, 4.12)	*p* = 0.006^**^
IR MCL (mm)	11.73 (11.00, 12.46)	9.75 (9.02, 10.49)	*p* < 0.001^***^
IR MCW (mm)	6.61 (5.83, 7.40)	5.04 (4.26, 5.83)	*p* < 0.001^***^
LG MCL (mm)	15.14 (14.05, 16.24)	15.34 (14.25, 16.44)	*p* = 0.690
LG MCW (mm)	4.99 (4.55, 5.43)	4.65 (4.21, 5.08)	*p* = 0.068
OF MCL (mm)	37.99 (36.31, 39.67)	36.82 (35.14, 38.50)	*p* = 0.006^**^
OF MCW (mm)	33.55 (32.38, 34.73)	32.00 (30.82, 33.17)	*p* < 0.001^***^
DWI			
IR ADC(×10^−6^ mm^2^/s)	1632.07 (1554.32, 1709.81)	1545.05 (1467.30, 1622.79)	*p* = 0.010^*^
MAGIC			
IR T1 (ms)	1223.73 (1033.36, 1414.09)	926.09 (735.73, 1116.45)	*p* < 0.001^***^
IR T2 (ms)	98.84 (86.42, 111.26)	90.55 (78.13, 102.96)	*p* = 0.066
IR PD (pu)	82.09 (77.02, 87.17)	69.23 (64.16, 74.31)	*p* < 0.001^***^
LG T1 (ms)	885.45 (823.14, 947.76)	845.80 (783.49, 908.11)	*p* = 0.126
LG T2 (ms)	77.64 (74.07, 81.20)	77.30 (73.73, 80.86)	*p* = 0.846
LG PD (pu)	80.09 (77.81, 82.36)	80.74 (78.47, 83.02)	*p* = 0.690
OF T1 (ms)	487.57 (477.64, 497.49)	474.48 (464.55, 484.40)	*p* = 0.035^*^
OF T2 (ms)	114.05 (112.06, 116.04)	116.11 (114.12, 118.10)	*p* = 0.035^*^
OF PD (pu)	112.88 (109.88, 115.87)	110.92 (107.93, 113.92)	*p* = 0.325
IDEAL-IQ			
IR FF (%)	11.59 (8.80, 14.38)	14.80 (12.02, 17.59)	*p* = 0.008^**^
LG FF (%)	17.48 (13.72, 21.24)	18.20 (14.44, 21.96)	*p* = 0.466
OF FF (%)	82.93 (81.61, 84.26)	86.09 (84.76, 87.41)	*p* < 0.001^***^

CAS, Clinical Activity Score; BCVA, best-corrected visual acuity; IOP, Intraocular pressure; DWI, diffusion weighted imaging; ADC, apparent diffusion coefficient; IR, inferior rectus; MCL, maximum coronal length; MCW, maximum coronal width; LG, lacrimal gland; OF, orbital fat; T1, T1 mapping; T2, T2 mapping; PD, proton density; FF, fat fraction; MAGIC, magnetic resonance image compilation; IDEAL-IQ, iterative decomposition of water and fat with echo asymmetry and least-squares estimation.

*p < 0.05, **p < 0.01, ***p < 0.001.

Hertel proptosis and the CAS decreased after treatment, from 18.78 to 16.85 mm (*p* < 0.001) and from 2.80 to 1.41 (*p* < 0.001), respectively. BCVA showed a borderline improvement (*p* = 0.055), whereas IOP was unchanged (*p* = 0.789). On morphologic MRI, proptosis decreased (21.69 to 18.88 mm, *p* < 0.001) with reductions in IR size, as reflected by decreases in MCL and MCW (both *p* < 0.001), and reductions in OF dimensions (OF MCL *p* = 0.006; OF MCW *p* < 0.001). LG dimensions did not change significantly (LG MCL *p* = 0.690; LG MCW *p* = 0.068). On DWI, the ADC of the IR decreased (*p* = 0.010). On MAGiC, IR T1 and PD decreased (both *p* < 0.001), whereas IR T2 did not reach statistical significance (*p* = 0.066). For OF, T1 decreased and T2 increased (both *p* = 0.035), while PD remained stable (*p* = 0.325). On IDEAL-IQ, FF increased in both the IR and OF (*p* = 0.008 and *p* < 0.001, respectively), whereas LG FF and LG MAGiC parameters showed no significant changes (all *p* > 0.05).

Collectively, teprotumumab was associated with concordant clinical improvement and selective structural and compositional changes in IR and OF, whereas LG measures and several ocular parameters remained largely unchanged.

### 3.2 Changes in laboratory and patient-reported outcomes after teprotumumab

Pre-treatment and post-treatment laboratory indices and GO-QOL are summarized in Table 3. Pre–post differences were assessed using paired t tests or Wilcoxon signed-rank tests, as appropriate.

**Table 3:** Changes in laboratory parameters and GO-QOL scores before and after teprotumumab treatment (*n* = 22).

Parameters	Pre-treatment (*n* = 22)	Post-treatment (*n* = 22)	*p*-value
Thyroid function			
T3 (nmol/L)	2.17 (1.63, 2.71)	2.05 (1.82, 2.33)	*p* = 0.726
T4 (nmol/L)	105.65 ± 24.61	103.44 ± 16.49	*p* = 0.736
FT3 (pmol/L)	5.71 ± 1.80	5.40 ± 1.14	*p* = 0.455
FT4 (pmol/L)	16.22 ± 5.41	16.28 ± 3.31	*p* = 0.966
TSH (mIU/L)	1.05 (0.01, 3.59)	1.79 (1.02, 2.83)	*p* = 1.000
TG (ng/mL)	65.00 (36.88, 100.88)	35.25 (20.12, 119.50)	*p* = 0.014^**^
TG Ab (IU/mL)	19.00 (15.82, 24.20)	22.05 (19.88, 25.82)	*p* < 0.001^***^
TPO Ab (IU/mL)	32.20 (15.45, 79.97)	31.35 (14.70, 99.05)	*p* = 0.483
TRAb (IU/L)	7.89 (3.60, 23.65)	2.55 (1.52, 4.28)	*p* < 0.001^***^
Immunological parameters			
NK (%)	8.00 (4.00, 10.10)	9.60 (4.80, 10.10)	*p* = 0.652
NK (absolute) (cells/μL)	131.00 (87.00, 166.00)	165.00 (119.00, 266.00)	*p* = 0.203
CD3 (%)	71.39 ± 6.82	71.12 ± 6.78	*p* = 0.578
CD3 (absolute) (cells/μL)	1384.50 ± 374.36	1744.32 ± 470.18	*p* = 0.002^**^
CD4 (%)	41.55 (37.35, 45.50)	41.85 (38.32, 45.17)	*p* = 1.000
CD4 (absolute) (cells/μL)	794.23 ± 202.05	1009.18 ± 258.55	*p* = 0.002^**^
CD8 (%)	28.05 (23.25, 30.77)	28.35 (22.85, 31.980)	*p* = 0.321
CD8 (absolute) (cells/μL)	541.50 ± 197.05	675.27 ± 245.11	*p* = 0.002^**^
CD4/CD8	1.53 (1.25, 1.93)	1.54 (1.23, 2.01)	*p* = 0.305
CD19 (%)	15.90 (11.90, 23.40)	18.60 (14.00, 22.30)	*p* = 0.734
CD19 (absolute) (cells/μL)	270.00 (223.00, 428.00)	368.00 (318.00, 462.00)	*p* = 0.098
Other			
PTH (pmol/L)	4.91 ± 1.51	4.58 ± 1.60	*p* = 0.138
IGF1 (ng/mL)	166.83 ± 53.07	490.09 ± 103.75	*p* < 0.001^***^
GO-QOL (score, 0–100)			
Overall	54.55 ± 20.63	67.05 ± 20.68	*p* < 0.001^***^
Visual function	60.51 ± 19.42	71.88 ± 19.17	*p* < 0.001^***^
Appearance	48.58 ± 21.99	62.22 ± 22.37	*p* < 0.001^***^

T3, total triiodothyronine; T4, total thyroxine; FT3, free triiodothyronine; FT4, free thyroxine; TSH, thyroid-stimulating hormone; TG, thyroglobulin; TG Ab, thyroglobulin antibody; TPO Ab, thyroid peroxidase antibody; TRAb, thyrotropin receptor antibody; NK, natural killer cells. CD3/CD4/CD8/CD19 (%), percentages of CD3 + T cells, CD4 + T helper cells, CD8 + cytotoxic T cells, and CD19 + B cells; CD3/CD4/CD8/CD19 (absolute), corresponding absolute counts. PTH, parathyroid hormone; IGF-1, insulin-like growth factor 1; GO-QOL, Graves’ orbitopathy-specific quality of life questionnaire.

Data are presented as mean ± SD for normally distributed variables and median (Q1, Q3) for non-normally distributed variables. Pre- and post-treatment measurements were compared using paired t tests for normally distributed variables and Wilcoxon signed-rank tests for non-normally distributed variables. All tests were two-sided, and *p* < 0.05 was considered statistically significant. *p < 0.05, **p < 0.01, ***p < 0.001.

Thyroid hormone levels (T3, T4, FT3, FT4, and TSH) did not change significantly (all *p* > 0.05). In contrast, thyroid-related antibodies showed significant changes, with decreases in thyroglobulin (TG, *p* = 0.014) and thyrotropin receptor antibody (TRAb, *p* < 0.001), and an increase in thyroglobulin antibody (TG Ab, *p* < 0.001), while thyroid peroxidase antibody (TPO Ab) remained unchanged (*p* = 0.483). Immunological cell percentages were stable, including NK%, CD3%, CD4%, CD8%, and CD19% (all *p* > 0.05), whereas absolute counts of CD3+, CD4+, and CD8 + T cells increased (all *p* = 0.002); CD19 absolute counts showed a nonsignificant trend (*p* = 0.098). PTH did not change (*p* = 0.138). IGF-1 increased markedly (*p* < 0.001). GO-QOL overall score and both subscales (visual function and appearance) improved significantly (all *p* < 0.001).

In summary, thyroid hormone levels and most immune cell proportions remained stable, while selected thyroid antibodies, IGF-1, and GO-QOL improved after treatment.

### 3.3 Associations between clinical response and MRI parameter changes

**Figure 4:**
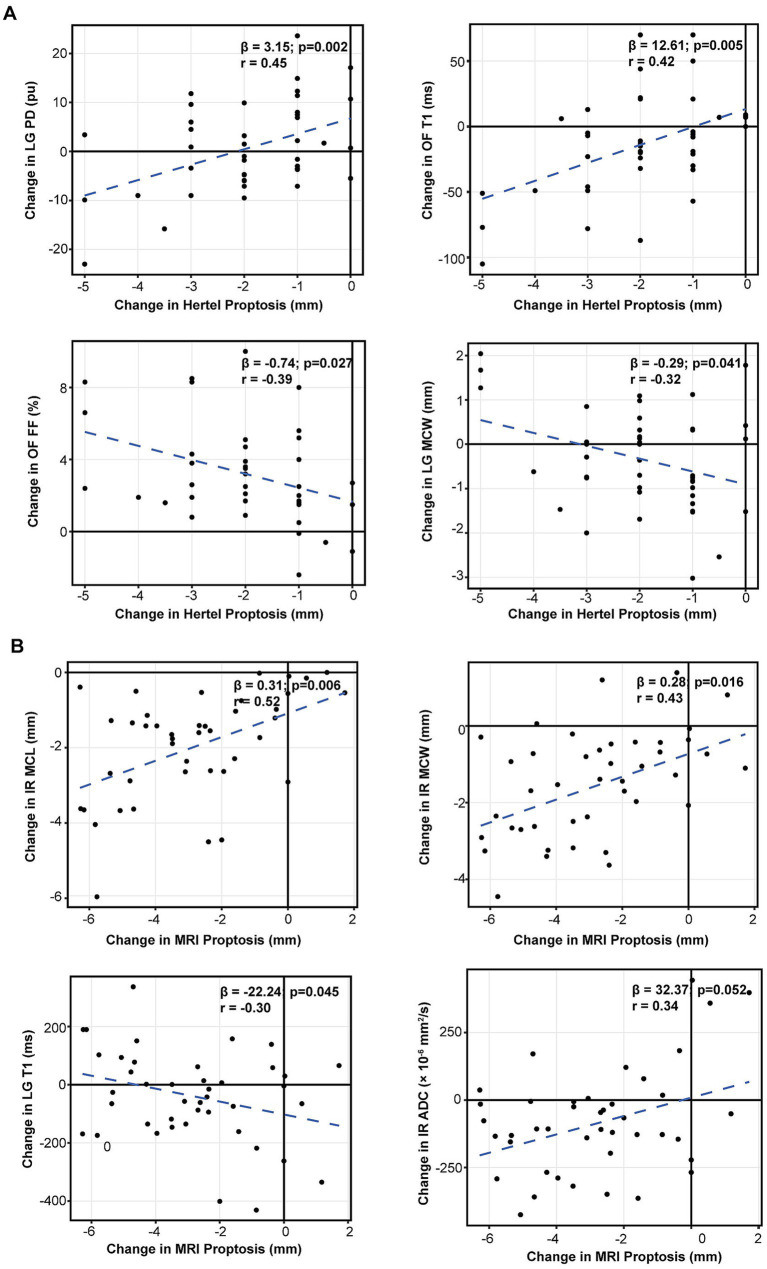
Associations between proptosis change and MRI parameter changes. *Δ* values were defined as post-treatment minus pre-treatment. Associations were evaluated using linear mixed-effects models with a subject-specific random intercept, and MRI *p* values were adjusted using the Benjamini–Hochberg procedure. **(A)** Δ Hertel proptosis versus ΔLG PD, ΔOF T1, ΔOF FF, and ΔLG MCW. **(B)** ΔMRI proptosis versus ΔIR MCL, ΔIR MCW, ΔLG T1, and ΔIR ADC. *β*, *r*, and adjusted *p* values are shown in each panel. IR, inferior rectus; LG, lacrimal gland; OF, orbital fat; PD, proton density; FF, fat fraction; ADC, apparent diffusion coefficient; MCL, maximum coronal length; MCW, maximum coronal width.

**Figure 5:**
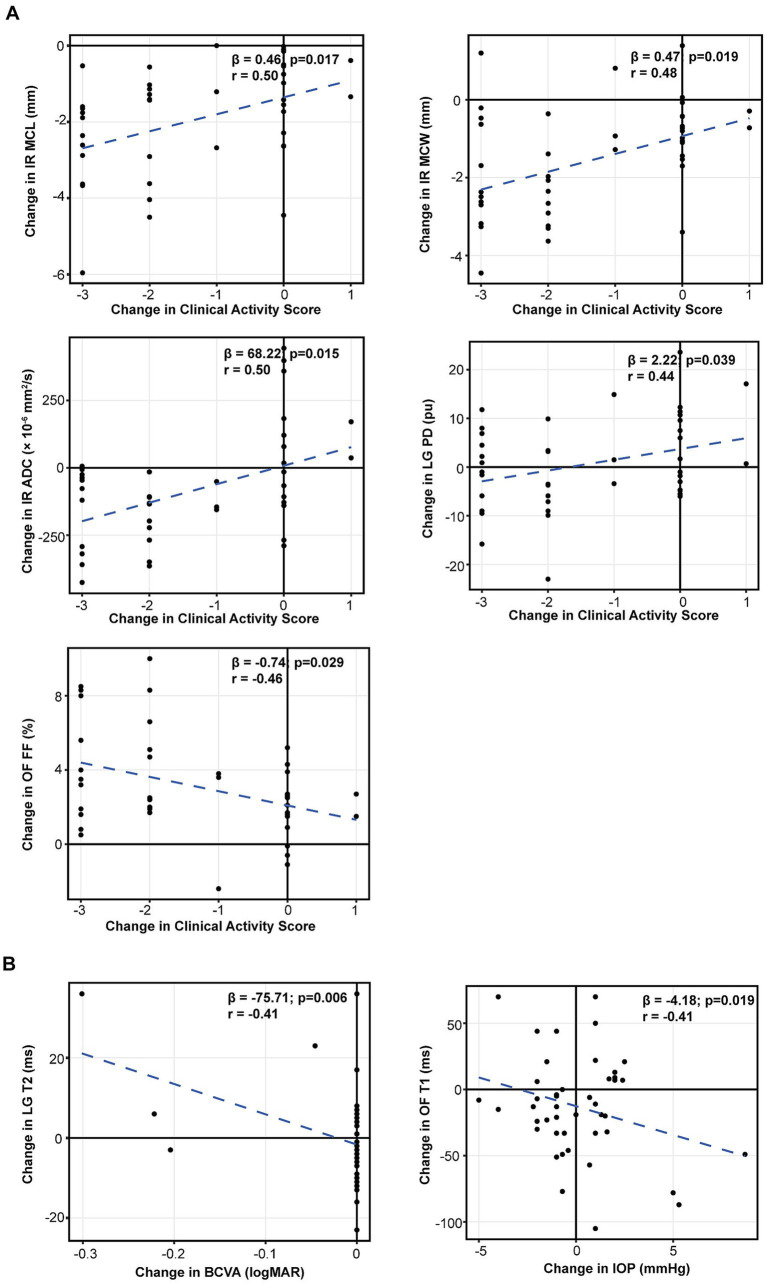
Associations between clinical outcome changes and MRI parameter changes. Δ values were defined as post-treatment minus pre-treatment. Associations were evaluated using linear mixed-effects models with a subject-specific random intercept, and MRI *p* values were adjusted using the Benjamini–Hochberg procedure. **(A)** ΔCAS versus ΔIR MCL, ΔIR MCW, ΔIR ADC, ΔLG PD, and ΔOF FF. **(B)** ΔBCVA (logMAR) versus ΔLG T2 and ΔIOP versus ΔOF T1. *β*, *r*, and adjusted *p* values are shown in each panel. CAS, Clinical Activity Score; BCVA, best-corrected visual acuity; IOP, intraocular pressure; IR, inferior rectus; LG, lacrimal gland; OF, orbital fat; ADC, apparent diffusion coefficient; MCL, maximum coronal length; MCW, maximum coronal width; PD, proton density; FF, fat fraction.

Changes were defined as post-treatment minus pre-treatment values (*Δ*), and associations were assessed using linear mixed-effects models with Benjamini–Hochberg–adjusted *p* values. Δ Hertel proptosis showed mild-to-moderate associations with Δ lacrimal gland proton density (ΔLG PD; *β* = 3.15, *r* = 0.45, adjusted *p* = 0.002) and Δ orbital fat T1 (ΔOF T1; *β* = 12.61, *r* = 0.42, adjusted *p* = 0.005), and inverse associations with *Δ* orbital fat fraction (ΔOF FF; *β* = −0.74, *r* = −0.39, adjusted *p* = 0.027) and Δ lacrimal gland maximum coronal width (ΔLG MCW; *β* = −0.29, *r* = −0.32, adjusted *p* = 0.041) (Figure 4A). *Δ*MRI proptosis was significantly associated with inferior rectus remodeling, including ΔIR maximum coronal length (ΔIR MCL; *β* = 0.31, *r* = 0.52, adjusted *p* = 0.006) and ΔIR maximum coronal width (ΔIR MCW; *β* = 0.28, *r* = 0.43, adjusted *p* = 0.016), and was inversely associated with Δ lacrimal gland T1 (ΔLG T1; *β* = −22.24, *r* = −0.30, adjusted *p* = 0.045), whereas the association with ΔIR apparent diffusion coefficient (*Δ*IR ADC) did not reach statistical significance (*β* = 32.37, *r* = 0.34, adjusted *p* = 0.052) (Figure 4B). *Δ* Clinical Activity Score (CAS) showed moderate associations with changes in IR size (ΔIR MCL: *β* = 0.46, *r* = 0.50, adjusted *p* = 0.017; ΔIR MCW: *β* = 0.47, *r* = 0.48, adjusted *p* = 0.019) and ΔIR ADC (*β* = 68.22, *r* = 0.50, adjusted *p* = 0.015), and additional associations with ΔLG PD (*β* = 2.22, *r* = 0.44, adjusted *p* = 0.039) and ΔOF FF (*β* = −0.74, *r* = −0.46, adjusted *p* = 0.029) (Figure 5A). In secondary analyses, ΔBCVA (logMAR) was inversely associated with Δ lacrimal gland T2 (ΔLG T2; *β* = −75.71, *r* = −0.41, adjusted *p* = 0.006), and Δ intraocular pressure (IOP) was inversely associated with ΔOF T1 (*β* = −4.18, *r* = −0.41, adjusted p = 0.019) (Figure 5B).

### 3.4 Associations between laboratory changes and clinical outcomes

To complement these eye-level analyses, we next evaluated whether patient-level changes in laboratory measures were associated with changes in CAS and GO-QOL. Spearman’s correlation was used to assess associations between changes in laboratory measures and changes in clinical outcomes. Changes in TRAb and IGF-1 were positively correlated with change in the CAS (TRAb: *r* = 0.366, *p* = 0.024; IGF-1: *r* = 0.426, *p* = 0.048). Changes in TG, TG Ab, and absolute T-cell counts (CD3, CD4, and CD8) were not correlated with CAS change (all *p* > 0.05). No significant correlations were observed between laboratory changes and change in QOL (all p > 0.05) (Table 4). Taken together, only *Δ* TRAb and Δ IGF-1 were associated with ΔCAS, whereas other laboratory indices and all associations with ΔGO-QOL were not significant.

**Table 4:** Spearman’s correlation between changes in laboratory measures and changes in CAS and GO-QOL.

Parameters and analysis	Change in CAS	Change in QOL
TRAb	*r* = 0.366, *p* = 0.024^***^	*r* = 0.277, *p* = 0.212
IGF1	*r* = 0.426, *p* = 0.048^***^	*r* = −0.166, *p* = 0.461
TG	*r* = −0.161, *p* = 0.473	*r* = −0.041, *p* = 0.856
TG Ab	*r* = 0.001, *p* = 0.998	*r* = 0.171, *p* = 0.446
CD3 (absolute) (cells/μL)	*r* = 0.105, *p* = 0.643	*r* = −0.192, *p* = 0.391
CD4 (absolute) (cells/μL)	*r* = 0.036, *p* = 0.873	*r* = −0.192, *p* = 0.391
CD8 (absolute) (cells/μL)	*r* = 0.085, *p* = 0.708	*r* = −0.146, *p* = 0.516

*r* refers to Spearman’s *r* values, while *p* refers to *p* (two-tailed).

## 4 Discussion

In this real-world study of Chinese patients with TED, we evaluated the clinical and imaging response to teprotumumab N01 Injection (SYCUME®) with a focused assessment of the IR, one of the most commonly and early involved EOMs in TED. Treatment was associated with meaningful improvements in proptosis and disease activity, accompanied by structural and quantitative MRI changes, particularly in IR dimensions and diffusion-related metrics. Moreover, IR-focused metrics showed consistent associations with clinical change, particularly IR MCL, IR MCW and diffusion-derived IR ADC, supporting their potential utility as imaging correlates of treatment response in routine practice.

Our findings indicate that teprotumumab N01 was associated with improvement in proptosis and disease activity, as reflected by reductions in Hertel measurements and CAS. These results align with prior randomized trials and subsequent real-world reports of teprotumumab demonstrating reductions in proptosis and inflammatory manifestations in TED (15–20). The magnitude of response should be interpreted in the context of baseline heterogeneity in this cohort, including variation in disease duration and the distribution of baseline activity, which may differ from trial populations (10, 14). Improvements were also observed in some orbits with lower baseline activity, although this observation requires confirmation in larger prospective cohorts. CAS is an ordinal composite outcome with a restricted range at lower baseline activity, which may reduce its responsiveness to change in some patients. In this context, structural and quantitative MRI measures may complement CAS by providing objective tissue-level information (10, 21, 22).

EOM involvement in TED is often asymmetric and contributes substantially to functional impairment. We therefore prioritized the IR for detailed analysis. The IR is commonly involved in TED, and enlargement of the IR is clinically relevant because it can contribute to motility restriction and orbital crowding (23, 24). Within this *a priori* IR-focused framework, IR morphologic measures showed treatment-associated changes and were consistently associated with clinical outcomes in our cohort. Reductions in ΔMRI proptosis were associated with decreases in IR MCL and IR MCW, and ΔCAS showed similar associations with changes in IR dimensions. Beyond morphology, ΔIR ADC was associated with ΔCAS, whereas its association with ΔMRI proptosis did not reach statistical significance. A recent study showed that after teprotumumab treatment, IR and medial rectus (MR) volumes decreased significantly, whereas lateral rectus (LR) and superior rectus (SR) volumes did not. Total extraocular muscle volume declined, while globe volume remained stable (25). Consistent with these observations, our IR-focused analyses aligned with established associations between extraocular muscle enlargement and key TED manifestations, including proptosis and inflammatory activity. Overall, IR-focused measurements may serve as a feasible quantitative adjunct to clinical assessment when monitoring tissue-level response during teprotumumab treatment.

Beyond morphologic imaging, we incorporated quantitative MRI metrics to further characterize treatment-associated orbital tissue changes. On DWI, IR ADC decreased after therapy, and ΔIR ADC was associated with ΔCAS. Although ADC is not disease-specific, prior work indicates that it is sensitive to changes in tissue microstructure and water mobility in inflammatory and remodeling settings (26, 27). DWI has also been increasingly applied to the orbit and EOMs, including in TED (13, 22, 28–30).

Quantitative mapping further showed decreases in IR T1 and PD, while IR T2 showed a non-significant downward trend. In contrast to the significant decreases in IR T1, PD, and ADC, IR T2 showed only a downward trend and did not reach statistical significance (*p* = 0.066). Quantitative T2 is commonly interpreted as a marker of tissue water content, particularly extracellular or “free” water, and prior studies have linked EOM T2 metrics to inflammatory activity in TED (31). However, T2 is also influenced by tissue composition and microenvironmental heterogeneity, including fat infiltration and fibrosis (32). Even with fat suppression, residual fat signal, partial-volume effects, and shifts in water compartment balance can affect measured T2 (33). Taken together, the lack of a significant T2 decrease may indicate that treatment-related effects on free-water components were modest or temporally lagged, whereas changes captured by T1, PD, and ADC may reflect earlier or more prominent alterations in the macromolecular environment and water mobility. FF increased in both the IR and OF. These parameters provide quantitative information related to relaxation properties and tissue composition, which may vary with edema, fibrosis, and fatty infiltration (26, 30, 34). Collectively, these quantitative signals may indicate coordinated shifts in tissue composition during treatment response, although their biological interpretation remains inferential without histopathologic validation (32, 35, 36). In our cohort, IR-focused metrics, including *Δ* IR MCL, Δ IR MCW and Δ IR ADC, showed consistent associations with changes in proptosis and CAS. These findings suggest that IR-centered imaging measures warrant further evaluation as candidate correlates of clinical response. Prospective studies with standardized acquisition, segmentation, and inter-reader reproducibility assessments, together with multi-timepoint imaging, are needed to establish reproducibility, define clinically meaningful thresholds, and determine whether baseline metrics improve prediction of treatment response beyond conventional clinical assessment.

Systemic laboratory measures showed selective changes following treatment. Thyroid hormone levels remained largely unchanged, whereas several thyroid-related antibodies and IGF-1 changed significantly, and patient-reported GO-QOL scores improved. In correlation analyses, changes in TRAb and IGF-1 were associated with ΔCAS, while other laboratory measures were not. No significant associations were observed between laboratory changes and ΔGO-QOL.

Notably, IGF-1 increased after treatment in our cohort. Similar patterns have been described in the context of IGF-1R pathway modulation and may reflect physiological regulation rather than clinical worsening (37, 38). More broadly, systemic biomarkers do not necessarily parallel orbit-level disease activity in TED, and prior studies have reported variable relationships between TRAb and clinical activity measures (39–41). Overall, the observed laboratory–clinical associations were modest, whereas orbit-level imaging measures may more directly reflect local, treatment-associated tissue changes (42, 43).

This study has several limitations. The retrospective, single-center design and modest sample size limit generalizability and preclude robust subgroup analyses. Although eye-level modeling was appropriate for asymmetric disease and we used mixed-effects approaches to account for inter-eye correlation, residual confounding and selection bias cannot be excluded. Imaging was assessed at two timepoints, which limits characterization of temporal dynamics and may miss early or delayed tissue changes. Quantitative MRI measures may vary with acquisition settings and ROI definition, and reproducibility across imaging settings warrants further validation. A comprehensive evaluation of all extraocular muscles was beyond the scope of this study, and future work should assess whether the observed associations extend to other muscles.

From a clinical perspective, assessing treatment response during the teprotumumab course can be challenging, particularly when improvement is gradual or asymmetric between orbits. Our findings suggest that interval changes in IR morphology (MCL and MCW), together with selected quantitative MRI metrics associated with changes in proptosis and CAS, may serve as objective adjuncts to routine follow-up. Incorporating these imaging measures alongside standard assessments of proptosis and appearance-related quality of life may help contextualize early response and inform individualized management decisions, while emphasizing that imaging should complement rather than replace clinical evaluation. In addition, longer-term outcomes such as the need for subsequent orbital decompression surgery are clinically relevant and reflect real-world treatment burden.

Future multicenter prospective studies with standardized acquisition protocols and multi-timepoint follow-up are needed to validate these imaging correlates, establish reproducibility and clinically meaningful thresholds, and determine whether early MRI changes predict patient-centered outcomes. Such outcomes may include sustained proptosis reduction, improvements in motility and diplopia, gains in GO-QOL, and reduced rates of subsequent decompression surgery (17, 44). Overall, findings from this real-world Chinese cohort are consistent with clinical effectiveness of a domestically produced teprotumumab formulation and identify IR-focused morphologic and quantitative MRI measures as candidate correlates of treatment response.

## Data Availability

The raw data supporting the conclusions of this article will be made available by the authors, without undue reservation.
